# Health-Related Experiences of Geophagia Among Women of Reproductive Age in Tshwane District, Gauteng Province: An Exploratory Qualitative Study

**DOI:** 10.3390/healthcare12202090

**Published:** 2024-10-21

**Authors:** Mohora Feida Malebatja, Moreoagae Bertha Randa, Mpata Mathildah Mokgatle, Oluwafemi Omoniyi Oguntibeju

**Affiliations:** 1 Department of Public Health, School of Healthcare Sciences, Sefako Makgatho Health Sciences University, Pretoria Ga-Rankuwa 0208, South Africa; moreoagae.randa@smu.ac.za (M.B.R.); mathildah.mokgatle@smu.ac.za (M.M.M.); 2 Phytomedicine and Phytochemistry Group, Department of Biomedical Sciences, Faculty of Health and Wellness Sciences, Cape Peninsula University, Bellville 3575, South Africa; oguntibejuo@cput.ac.za

**Keywords:** health related, experiences, geophagia, diseases, women of reproductive age, iron deficiency, Tshwane District

## Abstract

Background/Objectives: Women of reproductive age are reported to be the largest population that practises geophagia. The short-term and long-term health related conditions originating from the practise of geophagia are often not understood by geophagic women of reproductive age. This study has explored the health-related experiences of geophagia among women of reproductive age of Tshwane District, Gauteng Province. Methods: This was a qualitative study design following inductive thematic content analysis. In-depth interviews and focus group discussions were used to collect data using an interview guide and audio record. Data collection took place in 2023. Results: Mental health disorders, pregnancy complications, appendicitis, cancers, breathing complications, fatigue, premature deaths, worms, piles, and nutrients deficiencies were reported as some of the health-related challenges and diseases experienced by women of reproductive age of Tshwane District, Gauteng Province resulting from the practise of geophagia. Conclusions: The practise of geophagia was associated with various negative health outcomes. The reproductive health statuses of women of reproductive age were negatively impacted by the practise of geophagia affecting maternal and childhood mortalities and morbidities.

## 1. Introduction

Geophagia is defined as the continuous cravings of earth materials such as clay, soft rock, sediments, and soil [1,2,3]. Geophagia is classified as pica, and mental health disorder according to the American Psychiatric Association’s Diagnostic and Statistical Manual of Mental Disorders, Fifth Edition (DSM-5) and the World Health Organization (WHO) Clinical descriptions and diagnostic requirements for ICD-11 mental, behavioural, and neurodevelopmental disorders, respectively [4,5]. Pica is defined as an eating disorder of non-nutritive, non-food substances over a period of at least one month [4]. Some of the risk factors of pica include stress, pregnancy, cultural factors, underlying mental health disorder, learned behaviour, and nutritional deficiencies [4,5].

This practise is common amongst women of reproductive age in various African countries such as Ghana, Nigeria, Cameroon, Zambia, Mozambique, and South Africa [6,7,8,9,10,11]. However, there are men, elderly people, and children who indulge in the practise of geophagia as well [3,12,13]. Approximately 84% of clay soil consumers in sub-Saharan Africa were reported to be pregnant women [3]. In countries such as Nigeria and Tanzania, the prevalence of geophagia amongst pregnant women was reported to be 50% and 46%, respectively [3]. A review of some studies found that in South Africa the practise of geophagia was prevalent in Free state, Gauteng, KwaZulu Natal, Eastern Cape, and Limpopo Province [14].The prevalence of geophagia amongst women of reproductive age is unknown globally, however, the prevalence of geophagia is recorded to be the highest across different societies worldwide among pregnant women and children [3].The prevalence of geophagia amongst pregnant women in Tshwane, where this study was conducted found that 54% of pregnant women practise geophagy [15]. Majority of women of reproductive age practised geophagia at some point in their lifetime [3,9,16,17,18,19]. The practise of geophagia negatively impacts on maternal and child health as that is stipulated under SDG 3 of sustainable development goals of 2030 [2].

There are various chemical elements in varying quantities such as sodium, zinc, manganese, calcium, iron, mercury, silver, arsenic, lead, cobalt, cadmium, chromium and vanadium that are present in different clay soils consumed by women of reproductive age through the practise of geophagia [2,6,20,21,22,23]. There are also microbes such as helminths that are contained in clay soil ingested by women of reproductive age [10,22,24,25]. However, the chemical and microbiological composition of clay soil eaten by women of reproductive age and the dangers and consequences of geophagia are often not known and understood by those who indulge in this practise [3,20,22,26].

The uptake and adherence of iron supplements, folic acids, and vitamin B12 amongst women of reproductive age, must be stressed further to prevent the development of various clinical features of geophagia [27,28,29,30,31]. Studies indicate that there is a need to educate women of reproductive age about their physiological and nutritional needs particularly when pregnant to prevent development of iron deficiency that usually manifest in a form of uncontrollable cravings of clay soil that lead to the practise of geophagia [17,31,32,33,34].

The practise of geophagia serves as a serious public health concern that is linked with maternal and childhood morbidities and mortalities [35,36,37,38]. Presently, there are interventions available to curb the development of iron deficiency during pregnancy [29,31], however, there are no present interventions to teach women of reproductive age and the entire population about the dangers and consequences of practising geophagia. A gap has been identified that women of reproductive age are not aware of the acute and chronic health effects of geophagic practise. This study aimed to explore the health-related experiences of geophagia among women of reproductive age in Tshwane District, Gauteng Province.

## 2. Materials and Methods

### 2.1. Study Area

This study was conducted in the public healthcare facilities in Tshwane District, Gauteng Province, South Africa particularly in the antenatal care and family planning units. Tshwane has the population estimation of about 3.4 million excluding the undocumented immigrants [39]. Tshwane is also known to be the administrative capital city of South Africa situated in Gauteng Province, South Africa. Gauteng Province is known to be the economic hub. Public healthcare facilities in Tshwane District located in areas such as Soshanguve, Ga-Rankuwa, Mamelodi, Mabopane, Hammanskraal, Phelandaba, and Pretoria CBD were targeted as study settings to cover various women of reproductive age, considering that public healthcare facilities cater for the larger populations as compared to private facilities. The family planning unit and antenatal care unit were acronymised to FPU and ANC, respectively, to distinguish the participants when presenting the study findings. The practise of geophagy amongst women of reproductive age is also prevalent and visible in similar locations in Tshwane. The languages that are mostly spoken in Tshwane District include Isizulu, Sepedi, Setswana, Tshivenda and Xitsonga. Tshwane is also dominated by black Africans, whites and mixed race. The populations residing in Tshwane include elderly people, women, men, youth, and children from different ethnicities, cultures and traditions. Tshwane is also reported to have a significant number of women of reproductive age who practice geophagy.

### 2.2. Study Design, Population, and Recruitment

An exploratory qualitative study was conducted to explore the health-related experiences of geophagia amongst women of reproductive age in Tshwane District, Gauteng Province. Women of reproductive age who were above 18 years and willing to participate, who were accessing the antenatal care and family planning units of Tshwane District, Gauteng Province were included in the study as participants. Women below the age of 18 years were not considered for inclusion because we did not want to include minors in this study, since most of them are not yet ready for family planning or pregnancy since this study was conducted in the antenatal care and family planning. As much as a 16 years-old female can give consent on their own to access certain services in the reproductive healthcare units, to include them in this study was going to require assent from their parents to be able to participate. Exclusion and inclusion criteria were also based on women of childbearing age who are below the age of 18 years, and women who confirmed that they have reached menopause, meaning only those who confirmed that they still have the capacity to conceive a child and were above 18 years of age were considered for inclusion. The researcher scheduled an appointment with unit managers in the antenatal care and family planning units to recruit women of reproductive age who were consulting. The purpose of the study, aim, objectives, and information leaflets were discussed in detail with the potential participants during recruitment. Participants who agreed verbally to form part of the study were requested to sign written informed consent forms.

### 2.3. Data Collection

Data collection commenced after receiving ethics approval from Sefako Makgatho Health Sciences University Ethics Committee (SMUREC/H/290/2023: PG) and Tshwane Research Committee. Permission to conduct the study was sought from healthcare facility managers, and unit managers. The researcher conducted the interviews using an interview guide that consists of open-ended questions and audio recording device. In-depth interviews and focus group discussions were conducted with women of reproductive age using an adapted interview guide to explore the health-related experiences of geophagia amongst women of reproductive age who were accessing antenatal care and family planning units in Tshwane District. Data collection took 3 months between September and December in 2023.

### 2.4. Sample Size and Sampling Technique

Purposive sampling method was used to sample women of reproductive age who were consulting in the antenatal care and family planning units of Tshwane District, Gauteng Province who are willing to participate in the study and above 18 years of age. Purposive sampling technique was followed to select women of reproductive age to be included in the study. This was the best suited sampling technique to choose and categorise potential participants that possess a similar characteristic. The sample size was determined by data saturation, which is the stage where participants are no longer providing new information. The researcher conducted 15 in-depth interviews and 7 focus group discussions and reached a sample size of n = 99. The rationale for conducting both focus group discussions and in-depth interviews was to explore the experiences of geophagia phenomenon among women of reproductive age in-depth.

### 2.5. Data Analysis

An audio recording device was used to record and store data during the interviews. A computer device was used to store the data transferred from the audio records to generate transcripts. Qualitative data collected during the in-depth interviews and focus group discussions were processed and analysed using NVivo software 14. The transcripts were edited, cleaned, and exported to NVivo software 14 to identify and arrange codes. Thematic content analysis steps were followed to analyse qualitative data to produce a codebook [40]. The transcripts were read repeatedly to identify categorical data, and patterns to generate codes. Following the n-vivo software, 315 codes were generated from the study transcripts for all settings and participants. The generated codes were reviewed, then similar topics were merged to develop themes and sub-themes to produce coherent, concise, non-repetitive, and logical data. The new themes were reviewed and added, which were used to present the findings in the report. To cater for this objective, 3 themes were developed to present the study findings. Three people served as coders, the researcher and co-supervisor and statistician. The researcher worked with co-supervisor to code data and compared their codes and themes with data of the statistician who served as a co-coder. Descriptives statistics were used to describe socio-demographics data of women of reproductive age in Tshwane District, Gauteng Province.

### 2.6. Trustworthiness

As this was a qualitative study, it was necessary to ensure the dependability, transferability, conformability and credibility of the research findings [41]. The researcher kept field notes for all phases of the study, to ensure dependability. Credibility was ensured through recording and transcribing verbatim when conducting both the in-depth interviews and the focus group discussions. This ensured the accuracy of the data collected from all the participants. A thick description of the methodology and procedures was employed in this study, to allow the application of the same methodology in different settings and with different populations to ensure transferability. Conformability was achieved through auditing to ensure the coherence of the themes, so that the results would truly reflect the ideas of the participants.

### 2.7. Ethical Considerations

Clearance and approval were obtained from the Sefako Makgatho Health Sciences University Research Ethics Committee (SMUREC/H/290/2023: PG). Permission to conduct the study in the public healthcare facilities was sought from the National Health Research database committee (Tshwane Research Committee), senior managers of the healthcare facilities, and unit managers in the antenatal care and family planning units of Tshwane District, Gauteng Province. Research ethical principles that were observed in the study included privacy, confidentiality, informed consent, protection of personal information, voluntary participation, and right to withdraw from the study at any given time without any penalization.

## 3. Results

### 3.1. Socio-Demographics Characteristics of Women of Reproductive Age

The sample size consisted of n = 99 women of reproductive age who accessed reproductive healthcare services in the antenatal care and family planning units in Tshwane District, Gauteng Province. Seventy-six pregnant women and twenty-three non-pregnant women participated in the study. The participants ranged from eighteen years to fifty-nine years in age. Seventy-nine of the participants were single, followed by eighteen (18) that were married. Sixty-seven of the participants had secondary education as their highest level of education. Seventy-nine of participants were unemployed, followed by thirty been employed (see Table 1).

### 3.2. Verbatim Quotations Supporting the Emergence of Main Themes and Sub-Themes Are Demonstrated Below

#### 3.2.1. Occurrence of Mental Health Disorders

Women of reproductive age specified the practise of geophagia as a mental health disorder. The participants indicated that the uncontrollable cravings of clay soil exposed them to the development of mental health disorders such as addiction and dependency syndrome (refer to Table 2). The views are demonstrated below:

*“I started eating soil when I was pregnant, so but then it became a habit and something I was doing continuously as I was addicted”. (41 years old, employed, divorced, women of reproductive age, FPU, P17).*

*“I started consuming soil continuously when I crave it, although many times I would eat clay soil when I have my own stress to deal with my problems. So, I would ingest clay soil to keep myself distracted from life problems and challenges. I need a daily dose of clay soil to keep my mind sane”. (22 years old, unemployed, single, pregnant woman, ANC, P17).*

*“People who eat soil have strong addiction. They are normally stressed and therefore they opt for eating soil to forget about their problems. They have underlying issues that are occurring in their lives, so when they are alone or bored they eat this clay soil” (A 27 years old, pregnant women, ANC, P15).*

#### 3.2.2. Experience Reproductive Health Challenges and Complications

Reproductive health complications and fertility challenges were reported as some of the negative health effects associated with the practise of geophagia (refer to Table 2). Women of reproductive age highlighted that they experienced complications during their pregnancies that led to maternal and child health challenges such as miscarriages, pre-contamination of the foetus, low birth weights, and premature deaths that were driven by the practise of geophagia. The views are illustrated below:

*“I experienced constipation and piles resulting from the practise of geophagia. My system got blocked, then I went for deep cleaning, and they found mud in my entire system. I ended up giving birth to my child prematurely due to soil addiction, which led to delivery through caesarean-section”. (52 years old, employed, widower, women of childbearing age, FPU, P18).*

*“Geophagia causes appendicitis, premature deaths of babies, and iron deficiency, I thought it was not a bad thing to practise geophagia. I knew the dangers of consuming soil as piles only”. (29 years old, unemployed, single, pregnant woman, ANC, P28).*

#### 3.2.3. Development of Severe Chronic Medical Conditions

The practise of geophagia was highlighted by participants as a dangerous practise leading to the development of serious chronic medical conditions such as severe constipation, cancers, iron deficiency, worm infestations, appendicitis, piles, fatigue, and breathing complications were reported as some of the health-related conditions linked with the practise of geophagia amongst women of reproductive age(see Table 2). The views are expressed below:

*“When you start eating soil you endanger yourself whereby you end up developing appendicitis. People do not think that the practice is harmful to their bodies. The consequences of geophagia practise are usually witnessed at a later stage”. (56 years old, employed, married, women of reproductive age, FPU, P19).*

*“I ate soil to the extent of developing iron deficiency presently. I was hospitalised for a long period (months) due to complications caused by soil eating habit. Clay soil sucked my blood, I nearly died because of eating soil. I decided to stop the practise of geophagia after staying in a hospital undergoing various surgeries”. (34 years old, employed, single, women of reproductive age, FPU, P10).*

*“I have developed worms in my body due to soil eating habit. I thought it was flesh/ muscles/fats on my body, but I realised it is worms resulting from soil addiction. I cannot even confirm if I have stopped eating soil.” (41 years old, employed, divorced, women of reproductive age, FPU, P17).*

*“I was sick because of the geophagic practise, they told me at the hospital that I have iron deficiency. I was struggling with constipation and piles as well, due to the practise of geophagia. I spent a lot of time at the hospital because of complications caused by clay soil eating habit” (22 years old, unemployed, single, pregnant woman, ANC, P25).*

## 4. Discussion

In general, the short-term and long-term health related conditions associated with the practise of geophagia, are often not clearly understood by people who practise geophagia [22,25,26]. Majority of geophagic women of reproductive age lack awareness when it comes to the harmful health effects associated with the practice of geophagia [3,20,42]. This study has explored the health-related experiences of geophagia among women of reproductive age in Tshwane District, Gauteng Province.

The women of reproductive age who participated in this study revealed that practising geophagia leads to addiction and dependency syndrome over a prolonged time due to continuous cravings of clay soil. Similar studies found that perpetual ingestion of clay soil leads to addiction and dependency syndrome [43,44,45]. Similarly, geophagia was classified as a mental and behavioural disorder by (WHO) clinical descriptions and diagnostic requirements for ICD-11 [5]. Many people tend to substance abuse when they are faced with stress and other life challenges which ultimately become their coping mechanism leading to mental health disorders. Clay soil is addictive on its own, hence the people who practise geophagia find it difficult to quit the practise.

The participants indicated that they witnessed pregnancy complications, miscarriages, premature deaths, pre-contamination of the foetus, and giving birth to low birth weights due to the practise of geophagia. Similar studies also reported that pregnancy complications and giving birth to babies with central nervous systems challenges and low birth weights were connected to the practise of geophagia [1,2,20,42,46]. The excessive consumption of trace elements present in clay soil eaten by pregnant women, has the ability to alter the neurological aspects of the fetus and unborn babies [23]. The practise of geophagia makes pregnant women to lose appetite of eating healthy food due to constant constipation, which hinders the unborn babies to absorb as many nutrients as possible leading in low birth weight.

The practise of geophagia is regarded as a risky behaviour that is associated with development of negative health challenges such as cancers, breathing complications, fatigue, severe constipation, anaemia, appendicitis, worm infestations, and piles as indicated by women of reproductive age who took part in this study. Similar studies confirm that the practise of geophagia is linked to cancer development, anaemia, and delay of brain development of children, and parasitic infections [1,7,10,20,23,24]. In addition, another study found that aneamia, constipation, abdominal pain, diarrhea, delay of labour, and helminths were the health risks related to the practise of geophagia [26]. Indeed, the practise of geophagia is risky due to the chemical and microbial content of clay soil that contains harmful substances and agents, that have potential to cause diseases such as cancer and appendicitis. One of the interventions that must be looked into is the establishment of geophagy health education awareness campaigns to mitigate the practise of geophagia among women of reproductive age. This will further assist in preventing the development of diseases that may be arising from the practise of geophagia.

## 5. Conclusions

It is concluded that the women of reproductive age who practise geophagia are at risk of developing mental health disorders such as addiction and dependency syndrome. Women of reproductive age witnessed the development of severe medical chronic conditions such as cancers, breathing complications, fatigue, severe constipation, anaemia, appendicitis, worm infestations, and piles as some of the acute and chronic diseases, originating from the practise of geophagia. It is thus concluded that the reproductive health statuses of women of reproductive age are often negatively impacted by the practise of geophagia affecting maternal and childhood mortalities and morbidities. Further research is required to explore this phenomenon.

## 6. Study Limitations and Strengths

The setting and time were considered as the study limitations that might have influenced the participants to not share their experiences comfortably out of fear of healthcare workers since the study was conducted in the public healthcare facilities. Following purposive sampling might have also served as a study limitation since the study only considered women of reproductive age who were consulting in the antenatal care and family planning units. Raising awareness about the negative impact of geophagia on human health served as the strength of this study, since qualitative studies gives provision to inform participants about a particular phenomenon of interest that forms part of the societal problems.

## Figures and Tables

**Table 1:** Participants demographics.

Variable	Frequency	Percentages
Geophagia practise		
Consumer	50	51%
Non-consumer	49	49%
Age		
(18–19)	4	4%
(20–29)	44	44%
(30–39)	42	42%
(40–49)	5	5%
(50–59)	4	4%
Pregnancy status		
Pregnant	76	77%
Non- Pregnant	23	23%
Marital Status		
Single	79	80%
Married	18	18%
Divorced	1	1%
Windowed	1	1%
Education status		
Primary education	11	11%
Secondary education	67	68%
College	13	13%
Tertiary education	8	8%
Employment status		
Employed	30	30%
Unemployed	69	70%

**Table 2:** The health-related experiences of geophagia according to the participants were summarised and demonstrated into three main themes and sub-themes.

Theme	Sub-Theme
Occurrence of mental health disorders	Addiction
	Dependency syndrome
Experience reproductive health challenges and complications	Fertility and pregnancy problems
	Low birth weight babies
	Maternal and child deaths
	Pre-contamination of the foetus
Development of severe chronic medical conditions	Appendicitis
	Iron deficiency
	Worm infestations
	Piles
	Cancers
	Respiratory complications
	Fatigue
	Severe constipation

## Data Availability

Data were provided with submission.
